# A novel neural network model of Earth’s topside ionosphere

**DOI:** 10.1038/s41598-023-28034-z

**Published:** 2023-01-24

**Authors:** Artem Smirnov, Yuri Shprits, Fabricio Prol, Hermann Lühr, Max Berrendorf, Irina Zhelavskaya, Chao Xiong

**Affiliations:** 1 Helmholtz Centre Potsdam - GFZ German Research Centre for Geosciences, Potsdam, Germany; 2 Institute of Physics and Astronomy, University of Potsdam, Potsdam, Germany; 3 Department of Earth, Planetary and Space Sciences, University of California Los Angeles, Los Angeles, CA USA; 4 Department of Navigation and Positioning, Finnish Geospatial Research Institute (FGI), National Land Survey of Finland (NLS), Kirkkonummi, Finland; 5 Institute for Solar-Terrestrial Physics, German Aerospace Center, Neustrelitz, Germany; 6 Institute of Informatics, Ludwig-Maximilians-University of Munich, Munich, Germany; 7 Department of Space Physics, College of Electronic Information, Wuhan University, Wuhan, China

**Keywords:** Atmospheric science, Space physics

## Abstract

The Earth’s ionosphere affects the propagation of signals from the Global Navigation Satellite Systems (GNSS). Due to the non-uniform coverage of available observations and complicated dynamics of the region, developing accurate models of the ionosphere has been a long-standing challenge. Here, we present a Neural network-based model of Electron density in the Topside ionosphere (NET), which is constructed using 19 years of GNSS radio occultation data. The NET model is tested against in situ measurements from several missions and shows excellent agreement with the observations, outperforming the state-of-the-art International Reference Ionosphere (IRI) model by up to an order of magnitude, especially at 100-200 km above the F2-layer peak. This study provides a paradigm shift in ionospheric research, by demonstrating that ionospheric densities can be reconstructed with very high fidelity. The NET model depicts the effects of numerous physical processes governing the topside dynamics and can have wide applications in ionospheric research.

## Introduction

Earth’s ionosphere is a partially ionized region of the upper atmosphere, spanning from 60 to $\sim$1000 km in altitude^1^. The ionosphere is driven by a large number of competing processes and represents a highly dynamic medium that can change substantially in a matter of several minutes. A high number of free electrons in the ionosphere affects the propagation of radio signals, including those of the Global Navigation Satellite Systems (GNSS)^2^. Around 80% of the ionospheric total electron content (TEC) comes from the part located above the F-layer peak, known as the topside ionosphere^3^. Therefore, it is critically important to have accurate models of electron density in the topside ionosphere.

There are several approaches to model electron density in the ionosphere. The physics-based simulations, which obtain numerical solutions of the fundamental equations describing the ionospheric plasma, require sophisticated modeling codes that include coupling with the neutral atmosphere and magnetosphere^4,5^. Running such simulations is computationally expensive and thus problematic for operational purposes. An alternative approach to model electron density is through empirical modeling, where the relation between the input and output variables is described based on the statistical representation of observations^6^. Two of the most prominent empirical models of the ionosphere are the NeQuick model^7,8^ and the International Reference Ionosphere (IRI) model^3,9,10^. These models mainly describe the climatology of the ionosphere and reproduce regular variations of the ionospheric parameters. It is worth noting that recent versions of the ionospheric models, including the IRI-2016, are becoming increasingly oriented toward weather-like predictions, compared to earlier climatological descriptions^11^. The empirical models of electron density typically use a layered structure of the ionosphere, with the layer-peak parameters serving as anchor points of the density profiles^6^. In particular, two of the most important parameters are the peak density of the F2 layer (NmF2) and the corresponding altitude of the peak (hmF2), which have received a lot of attention in literature and can be well reproduced by the existing empirical models. In the topside ionosphere however, the models exhibit notable discrepancies from observations due to the highly non-uniform data coverage both in terms of solar activity and, most importantly, in altitude^12–14^. Therefore, accurate topside modeling has remained a significant challenge.

Over the last 2 decades, the ionosphere has become a data-rich environment. One of the most efficient ways to make use of the vast amounts of data for empirical modeling is by applying machine learning (ML) techniques. In recent years, a number of ML-based electron density models in the Earth’s ionosphere have been developed^15–20^. Several models provide the F2-peak parameters^15,18^, while others reproduce three dimensional electron density distributions^16,17^. However, most existing ML-based models binned the data into spatial cells in terms of geographic latitude and longitude and thus do not provide continuous output^16,19,20^. Habarulema et al.^19^ noted that these models exhibit discontinuities at the borders of spatial cells and require either 3D interpolation or filtering to produce physically meaningful output. Furthermore, it has been noted that one of the main limitations of the existing machine learning models is their lack of validation through independent data sources^19^.

**Figure 1:**
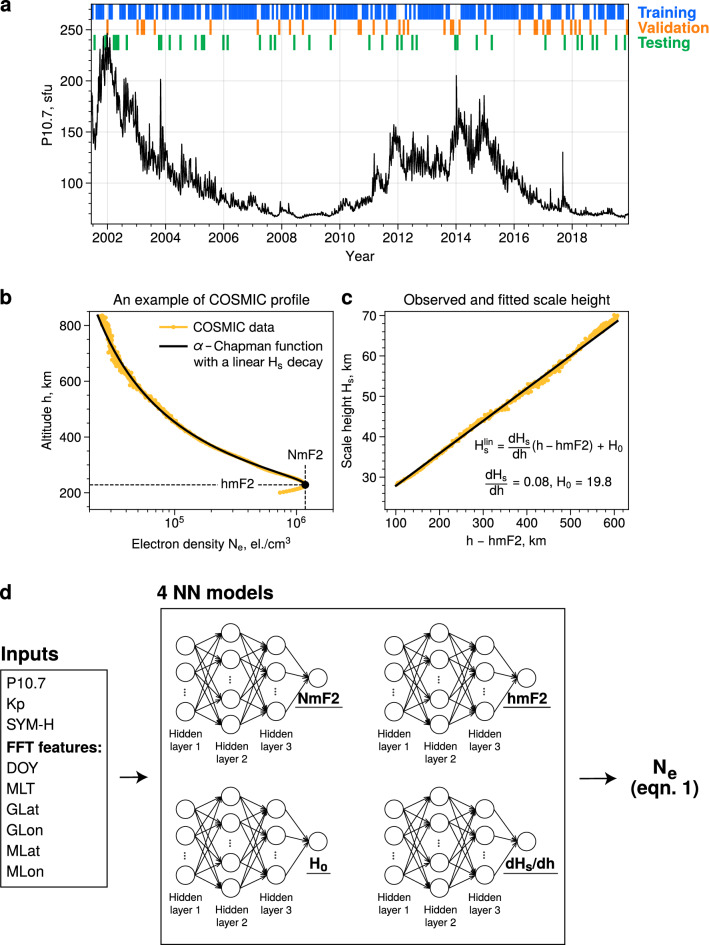
(**a**) Distribution of the P10.7 index and the data splitting; (**b**) An example of the COSMIC profile (orange) and the fitted data using the alpha-Chapman function with a linear decay of scale height with altitude (black); (**c**) Observed scale height and the linear fit; (**d**) Schematics of the model workflow.

In this study, we present an empirical model of electron density in the topside ionosphere based on Chapman functions with a linear dependence of scale height on altitude. Our model consists of 4 parameters, namely the electron density of the F2-peak (NmF2), the peak height (hmF2), and 2 parameters of the linear scale height decay (slope $\mathrm {dH_s/dh}$ and intercept $\mathrm {H_0}$), derived from 19 years of GNSS radio occultation (RO) data (see Fig. 1(a)). We use neural networks to model these parameters and develop a continuous model that yields highly accurate reconstructions of the topside ionosphere. We perform an extensive validation of the model on in situ data from three independent missions not used for the model training. The developed Neural network-based model of Electron density in the Topside ionosphere (NET) model is in a remarkable agreement with in situ measurements and outperforms the IRI-2016 model by up to an order of magnitude, especially at 100-200 km above the F2-layer peak and in local-winter hemispheres.

## Results

### Modeling 4 parameters of the linear alpha-Chapman function using neural networks

**Figure 2:**
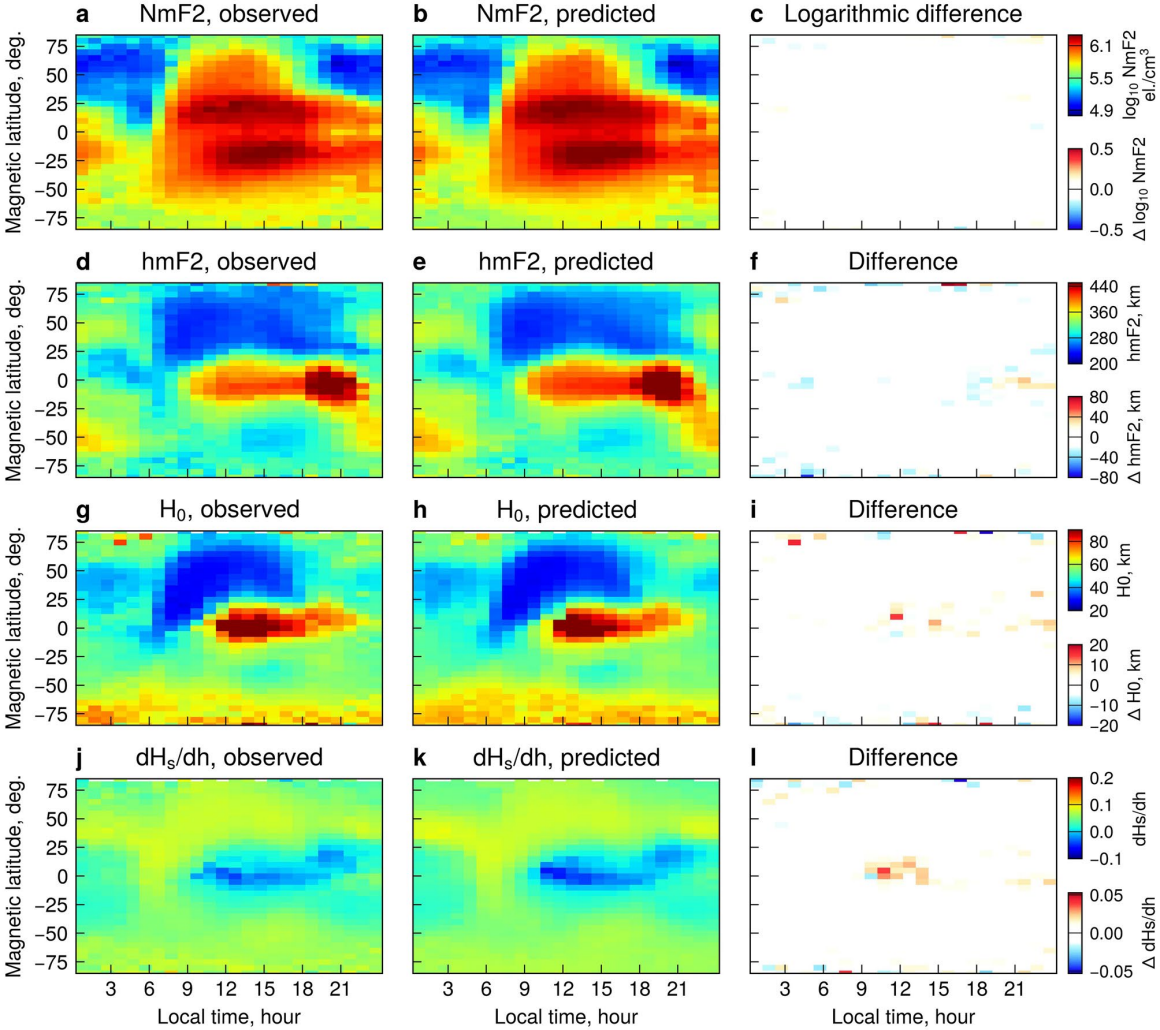
Maps of the four parameters observed by COSMIC and predicted using the NET model, binned by magnetic latitude and local time. The bins with <2 data points were removed. The data cover the time interval from 2013-11-11 until 2014-02-27, corresponding to D-season conditions, and are sampled from the validation and test sets.

The developed NET model consists of 4 sub-models reproducing parameters of the linear alpha-Chapman equation based on location, season, magnetic local time, and solar and geomagnetic activity (Fig. 1). It is, firstly, necessary to evaluate the ability of the model to recreate these four parameters correctly. A comparison between the values predicted by the NET model and Constellation Observing System for Meteorology, Ionosphere and Climate (COSMIC) observations on the training, validation and test sets (Fig. S1 in the Supplementary Information) yields that the model reproduces all of the parameters well, with correlation coefficient ranging from 0.8 for the $\mathrm {dH_s/dh}$ model up to 0.96 for the NmF2 model. Furthermore, the correlation coefficient values are nearly identical on the training, validation and test sets which indicates a very good generalization ability of the model and low degree of overfitting. We now move to analyzing whether the sub-models of each parameter preserve realistic structures of the ionosphere.

Figure 2 shows a comparison between the observed and predicted values of the four parameters based on 3 months of COSMIC data sampled from test and validation sets. The values are binned by 1 hour magnetic local time (MLT) and 5 degrees Magnetic Latitude (MLat). The interval in question covers the times from late November 2013 until end of February 2014, which correspond to the December solstice (D-season) conditions. NmF2 and hmF2 parameters describe the dynamic variability of the F2-peak, while the $\mathrm {H_0}$ and $\mathrm {dH_s/dh}$ interpreted together can be indicative of the processes in the topside. In particular, Prol et al.^21^ showed that $\mathrm {H_0}$ generally shows the electron content directly above the F2 peak, while changes in $\mathrm {dH_s/dh}$ account for electron density decay at higher altitudes. Specifically, higher $\mathrm {H_0}$ values correspond to the “thicker” profiles around the peak, while the increased $\mathrm {dH_s/dh}$ values indicate steepening of the profiles and slower decay at high altitudes.

#### Physical phenomena as seen in the NET model results

Several known regions, corresponding to different processes that govern the ionospheric dynamics around December solstices, can be identified in the results of NET (Fig. 2). NmF2 exhibits two crests of ionization around 15–25$^\circ$MLat in both hemispheres, separated by an electron density trough. This feature is known as the Equatorial Ionization Anomaly (EIA) (Fig. 2(a,b)); it usually develops after sunrise and shows a gradual decay after midnight^1,22^. The EIA formation can be explained as follows. The existence of zonal electric fields around the equator, where the magnetic field lines are nearly horizontal, gives rise to the vertical **E**$\times$**B** drift. During the daytime, the zonal electric fields are directed eastwards which leads to the upward transport of plasma by the **E**$\times$**B** drift. During sunrise, an increase in solar illumination ionizes the neutral particles in the thermosphere. These newly ionized particles are transported to higher altitudes by the **E**$\times$**B** drift, and an enhancement of hmF2 develops at $\sim 06$ MLT around the equator. Higher altitudes have lower recombination rates, and therefore the ionized particles pertain there for longer times and start diffusing downward under the gravity and pressure gradient forces. This diffusion is constrained by the magnetic field lines and leads to the formation of the two crests of ionization, and this is known as the equatorial fountain effect^22^. A global maximum of hmF2 is manifested around the equator at $\sim$19 MLT (Fig. 2(d,e)). This corresponds to the sunset hours, when the eastward electric fields exhibit pre-reversal enhancements (PRE)^23^ leading to large upward **E**$\times$**B** velocities which lift the F2 peak to even higher altitudes ($>460$ km). During the nighttime (21-06 hours MLT), the zonal electric fields reverse their direction and move westwards creating the downward **E**$\times$**B** transport. The F2-peak thus subsides to lower altitudes ($\sim 280$ km). Due to higher neutral densities and stronger recombination rates there, NmF2 starts slowly decaying exhibiting minima around 05 MLT (Fig. 2(a,b)).

Figure 2 corresponds to the D-season conditions with the local summer in the southern hemisphere. NmF2 exhibits a strong hemispheric asymmetry, with larger electron densities in the southern hemisphere, due to the Earth’s tilt and higher solar irradiation (Fig. 2(a,b)). Furthermore, hmF2 also shows a summer-winter asymmetry with values in the northern hemisphere lower by about 100 km compared to the southern hemisphere. Around the solstices, there are strong winds blowing from the summer into winter hemispheres at altitudes around the F2-peak^22^. In the D-season, the winds are directed from the southern into the northern hemisphere. These winds have a component parallel to the magnetic field and transport ionization upward in the local-summer hemisphere, while pushing the F2-peak downward in the local-winter hemisphere, resulting in the asymmetric structure of hmF2. Furthermore, one interesting feature in Fig. 2(g,h) is the increase in $\mathrm {H_0}$ at polar latitudes in the southern hemisphere, which indicates that electron density profiles are convex around the peak. This pattern shows little diurnal variation and likely comes from the fact that during solstices, polar regions in the local-summer hemispheres are sunlit at all MLTs due to absence of dark nights^21^. One also observes several regions of enhanced scale height gradient (Fig. 2(j,k)), namely two mid-latitude stripes ($\sim 40^\circ$ MLat in both hemispheres) which remain at all local times, and a sunrise peak ($\sim$05–07 MLT) around the magnetic equator. The sunrise peak is likely connected with the so-called morning overshoot of electron temperature. It happens due to energy exchange between the newly ionized photoelectrons and ambient electrons, which is more efficient in regions of low electron density. Once the ionization builds up, the resulting cooling decreases the temperature and the peak disappears at around 08 MLT^24^. As the temperature is related to scale height, this feature pertains in the scale height gradient. The $\mathrm {dH_s/dh}$ peaks at middle and high latitudes are likely due to the downward fluxes of protons injected into the topside from the plasmasphere^21^, and become more evident at low solar activity (see Supplementary Figure S3).

All of the above-mentioned processes are very well depicted by the NET model, as the differences between the NET predictions and COSMIC observations of the 4 parameters are close to zero at all magnetic latitudes and local times. This means that the NET model is capable of reproducing ionospheric dynamics caused by a wide variety of drivers, including the electrodynamic processes, neutral winds, field-aligned transport from the magnetosphere, etc. Furthermore, the combined interpretation of the four model parameters can yield insights into the physical processes that control the dynamics of the topside ionosphere.

**Figure 3:**
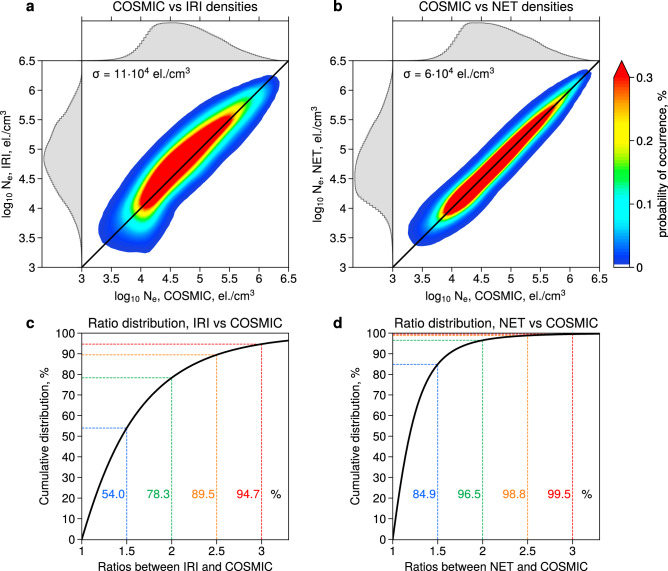
2D histograms of electron density observed by COSMIC on the test set versus those predicted by the IRI model (**a**), and the developed NET model (**b**). (**c**) Cumulative distribution of ratios between the IRI model and the COSMIC data on the test set; (**d**) Cumulative distribution of ratios between the developed NET model and the COSMIC data on the test set.

### Model testing on COSMIC electron densities

We now move to evaluating the model performance on COSMIC electron density values. Fig. 3 shows a comparison between electron density predictions by the IRI and NET models to the COSMIC data for the entire test set. In panel (a), we demonstrate the 2D histogram of electron densities observed by COSMIC and predicted by the IRI-2016 model with the topside specified by the NeQuick option. The IRI gives unbiased predictions for very low and very high electron densities, as these points generally lie close to the one-to-one correspondence line. However, for intermediate densities ($\sim 10^5$ el./cm$^3$) there is an overestimation of electron densities by the IRI, which results in a curved shape of a 2D probability distribution. Furthermore, the IRI model produces a close-to-gaussian distribution of electron density values, while the distribution of the COSMIC data appears more flat-top and is skewed to the right. The comparison of the NET predictions to COSMIC data is shown in Fig. 3(b). The corresponding 2D probability distribution is centered around the one-to-one line, which means that the model gives unbiased predictions in the topside ionosphere when compared to COSMIC data. The overall bias of the NET model on the test set is approximately 3 times smaller than for the IRI (–0.6$\cdot 10^4$el./cm$^3$ compared to $\sim 1.8\cdot 10^4$el./cm$^3$). Furthermore, in Fig. 3 one can see that the 2D probability distribution appears narrower for the NET model. To quantify the degree of spread of the 2D distributions, we evaluate the standard deviation of the difference between the observed and predicted electron densities. The standard deviation for the NET model is $6.5\cdot 10^4$el./cm$^3$, while for the IRI this value equals $11\cdot 10^4$el./cm$^3$, which is approximately 1.6 times larger. This indicates that the dynamics of electron density is on average captured better by the NET model. Another useful metric, often employed when evaluating the model predictions, is the ratio between the model predictions and observations. Figure 3(c,d) shows cumulative distributions of the ratios between the IRI and NET models to COSMIC data. We use the linear version of the ratio for values $>1$, while taking the inverted ratios if the values are $<1$. The cumulative distributions of the ratios, shown in Fig. 3(c,d), yield how often the model predictions lie within a given factor from the data. One can see that 96.5% of the time the NET electron densities lie within a factor of 2 from the observations, while the IRI predictions are within a factor of two 78.3% of the time. Moreover, most of the NET predictions (over 84.9%) are within a factor of 1.5 from the data, which is higher than for the IRI (54%).

**Figure 4:**
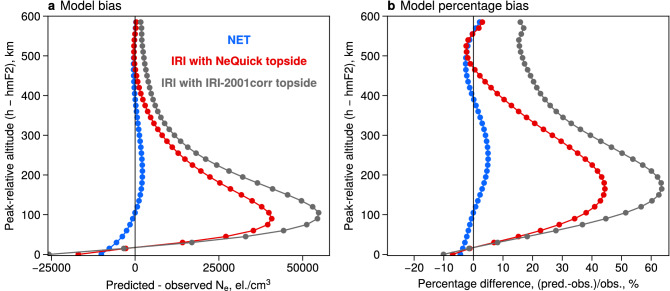
Median bias (**a**) and median percentage bias (**b**) versus altitude relative to the F2-peak, calculated on the test set of the COSMIC data. Biases of the developed NET model are plotted in blue. Vertical residuals of the IRI-2016 model are shown in red for the NeQuick topside option, and in grey for the IRI-2001corr topside shape.

The statistics shown in Fig. 3 combine all altitudes and locations corresponding to the test intervals of the COSMIC data. In order to investigate the performance of the developed NET model in more detail, we bin the data in several dimensions and evaluate the metrics locally, compared to the previous global comparisons. We, firstly, bin the model residuals by altitude relative to hmF2 (Fig. 4). It is of note that for this comparison we do not distinguish between different locations, and provide such binning separately in the Supplementary Figure S4. Figure 4(a) indicates that the IRI-2016 model with the NeQuick topside underestimates the F2-peak density, and overestimates electron densities in the topside. In particular, the strongest overestimation by the IRI, of up to 40.000 el./cm$^3$, comes from the region around 100 km above the F2 peak height. Similarly, the developed NET model also underestimates the peak densities, although the bias is roughly 1.5 times smaller than for the IRI. In the topside ionosphere, however, the vertical residuals of the NET model are significantly lower than for the IRI. This is also illustrated in Fig. 4(b) that shows the vertical percentage biases. The largest percentage error for the IRI is located at around 150-200 km altitude from the peak. This overestimation reaches >40%, while at higher altitudes, the residuals are smaller and converge to become almost unbiased at 500 km above hmF2. In case of the NET model, the residuals are very small at all altitudes, and do not exceed 5-7%. It is worth noting that in the region where the IRI-2016 exhibits the largest error, the NET model becomes almost unbiased and only shows deviations from the data in the order of several percent. In Fig. 4, we also demonstrate the median bias by the IRI model when the topside is given by the IRI-2001corr shape function, which is based on the early formulation of Bent et al.^25^. In Fig. 4b, one can see that at altitudes $\sim 150$ km above the peak, the bias is significantly higher for the IRI-2001corr topside option (>60%) than for the NeQuick ($\sim$40%).

The seasonal behavior of the models is investigated in Fig. S4 in the Supplementary Information. The figure shows that the NET model gives largely unbiased predictions for all seasons, as the average ratios between the model predictions and COSMIC observations are very close to 1. In case of the IRI, the model overestimates densities in the local-winter hemispheres for December and June solstices, and overestimates the equatorial ionization anomaly crests during equinoxes. This overestimation corresponds to large values of skill score (up to 80%) and highlights the regions where the developed NET model most significantly outperforms the IRI (see the Supplementary Figure S4). Overall, these results demonstrate that the NET model gives unbiased and highly accurate predictions of electron density in the topside ionosphere, remains unbiased for all seasons, and consistently outperforms the IRI model by up to an order of magnitude, especially around 200 km above hmF2 and during the local winters.

### Model testing on independent observations

**Figure 5:**
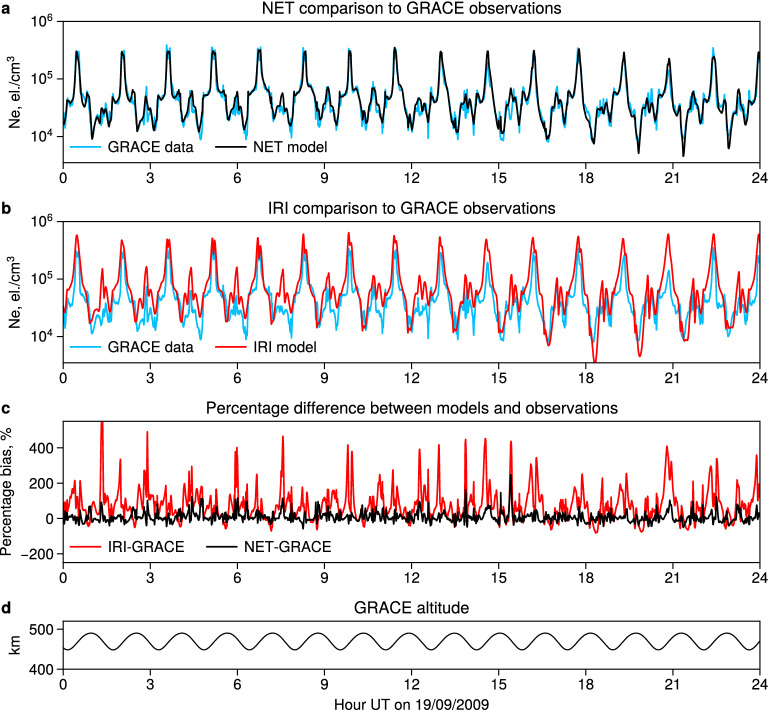
GRACE/KBR densities (shown in blue), compared to the NET predictions (panel** a**), and IRI predictions (subplot** b**) on 19 September 2009 (an example from the test interval). The percentage differences between the models and observations are given in panel (**c**). The GRACE altitude is shown in panel (**d**).

In the previous subsection, the model performance was analyzed on electron densities from the COSMIC mission. Due to the fact that the NET model was trained on COSMIC data, it is crucial to perform an additional validation on the fully independent data sources. The purpose of such validation is to ensure that the model not only reproduces the COSMIC line-of-sight profiles which may smear out some of the ionospheric structures but is also capable of resolving the finer morphology of the topside ionosphere. Therefore, in this section we test the model on 3 completely independent missions, namely, the Gravity Recovery And Climate Experiment (GRACE), Challenging Minisatellite Payload (CHAMP) and Communications/Navigation Outage Forecast System (C/NOFS). These missions provide data of the highest quality and have been used in a variety of ionospheric studies, both for empirical modeling and for case studies analyzing specific space weather events.

An example of the model comparison to GRACE K-Band Ranging (KBR) data for one of the days corresponding to equinoctial conditions is shown in Fig. 5. Note that this day was not used for model training and belongs to the test set. In panel (a), we show electron densities observed by GRACE and predicted by the NET model. The model values are in excellent agreement with the observations, as the NET model correctly captures both the high electron density values corresponding to equatorial latitudes and the low densities typically occurring at higher latitudes. Figure 5(b) gives a comparison between the IRI predictions and GRACE-KBR observations. It is evident that the IRI overestimates electron densities in the equatorial region. This is in good agreement with our previous comparison between the IRI model and COSMIC data (Supplementary Figure S4), which showed that the IRI overestimates the EIA crests by approximately a factor of 2. Furthermore, Fig. 5(b) also shows that the IRI strongly overestimates the regions of low densities, which correspond to higher latitudes. In fact, the density depletions shown in Fig. 5 are due to the midlatitude ionospheric trough, which is known to be challenging region for empirical modeling of the ionosphere. Figure 5(c) gives the percentage bias for both models. One can see that the IRI model overestimates electron densities by up to 400% in the polar regions, and that the model is in general biased towards overestimation. The NET model, on the other hand, shows much lower percentage bias which is centered at zero. It should be noted that throughout the period demonstrated in Fig. 5, GRACE altitude was about 450-480 km, which is approximately 100-150 km above the F2 peak. In Fig. 4, this region was highlighted as the most problematic for the IRI with an overestimation of the COSMIC densities by almost 50%. The percentage bias demonstrated in Fig. 5(c) agrees well with our findings in Fig. 4, as the IRI tends to overestimate the GRACE-KBR electron densities in the same way as COSMIC. The NET model shows mostly unbiased predictions along the GRACE altitude.

**Table 1:** Metrics for comparisons between NET and IRI models and observations on 3 independent missions.

Metric	CHAMP-PLP	GRACE-KBR	C/NOFS-CINDI
Median bias, NET, [el./cm$^3$]	11.209	460	–988
Median bias, IRI, [el./cm$^3$]	34.021	8.708	14.422
Standard deviation, NET, [el./cm$^3$]	182.585	127.390	101.820
Standard deviation, IRI, [el./cm$^3$]	218.160	165.048	161.739
Median log bias, NET	0.04	0.003	–0.01
Median log bias, IRI	0.1	0.05	0.12
% of values within a factor of 2, NET	88	89	91
% of values within a factor of 2, IRI	78	79	75
Correlation, NET, [%]	92	92	92
Correlation, IRI, [%]	87	85	86
Skill score of NET over IRI, [%]	30	40	60

In the Supplementary information, we demonstrate additional examples of the model testing. Figure S5 gives an example of a 27 day period from the test set, showing the satellite passes of GRACE and predictions by the NET and IRI models. It can be seen that the NET model reproduces the ionospheric structures very well, and is even able to capture electron densities in the midlatitude ionospheric trough, which has been shown to be a traditional challenge for ionospheric modeling^26^. The metrics evaluated on the test set show that the NET model predictions are in very good agreement with observations (Table 1), as $\sim$90% of the NET electron densities lie within a factor of 2 from the measurements based on CHAMP, GRACE and C/NOFS missions. Furthermore, the NET model exhibits very low values of bias, e.g., $\sim -10^3$el./cm$^{3}$ for C/NOFS, which is approximately an order of magnitude smaller than for the IRI. This indicates that the developed NET model is not only capable of reproducing the COSMIC data which were used for model training but also yields highly accurate predictions on completely independent measurements by 3 instruments operating on different observational principles.

## Discussion

The Earth’s ionosphere is a region of paramount importance for a variety of scientific and industrial applications. In particular, the ionospheric delays are one of the largest error sources for GNSS navigation. Since these delays are proportional to the total electron density along the ray path, it is crucial to have accurate and reliable models of ionospheric electron densities. The bottomside ionosphere accounts for around 20% of the total electron content, while the main contribution to the TEC magnitudes comes from the topside^3^. Modeling of the topside ionosphere has remained a significant challenge, due to data sparsity in both spatial and temporal domains. As a result, the existing ionospheric models show substantial differences from the observations, evident from comparisons to both in situ measurements and integrated TEC magnitudes^13,14,27^. In this study, we use radio occultation data, which constitute a large data set of electron densities with uniform coverage of the topside, and develop a continuous NN-based model of electron density in the topside ionosphere. The developed NET model yields highly accurate predictions at all altitudes, locations, seasons and solar activity levels and outperforms the international standard of the ionosphere – the IRI – by up to 80$\%$.

The comparison of the IRI electron density predictions to COSMIC Electron Density Profiles (EDPs) revealed the typical structure of the vertical biases of the IRI in the topside ionosphere. In particular, our results show that the most significant deviations ($\sim 40\%$) of IRI-2016 with the NeQuick topside from the data are located around 100–200 km above the F2-peak. At higher altitudes, the residuals decrease to the order of several percent. The vertical residuals of the IRI model, shown in Fig. 4, are likely due to the shape function used to parametrize the topside in the NeQuick model. The NeQuick treats the semi-Epstein scale height as a function of three empirical parameters, namely $\hbox {H}_0$, which shows the scale height around hmF2, *g*, representing the gradient of scale height near the peak, and *r* that controls the asymptotic behavior of the profiles at infinity (for details, see e.g. Nava et al.^7^). Recent studies have highlighted the rigidity of this parametrization, which is due to the fact that two of the parameters are kept constant ($r=0.125$ and $g=100$) while $H_0$ is modeled as a function of the bottomside profile thickness^28–30^. The processes that control the bottomside dynamics are different from those in the topside, and this can yield inaccuracies in the $H_0$ parametrization. Themens et al.^29^ demonstrated that the NeQuick tends to overestimate $\mathrm {H_0}$ values during the solar minimum conditions and underestimate $\mathrm {H_0}$ during the solar maximum. These results go well with our comparison to COSMIC data shown in Fig. 4. When $\mathrm {H_0}$ is overestimated, the overall profile shape becomes too convex compared to the data, and the most significant deviations would be found at round 150-200 km above hmF2 (see Fig. 6 in Themens et al.^29^). Furthermore, Pignalberi et al.^28^ recently demonstrated that the topside gradient *g*, which has a fixed value in the NeQuick, also exhibits strong variations based on geophysical conditions. The study indicated that the NeQuick parametrization could by improved by creating additional sub-models of $H_0$, *r* and *g*. In the current study, we use a flexible parametrization of the topside scale height, allowing the scale height gradient to vary with location, local time, season, solar and geomagnetic activity, and therefore the NET model remains unbiased around 100–200 km above hmF2. Another possible reason for the vertical residuals seen in Fig. 4 is a potential overestimation of hmF2 by the IRI. Bilitza et al.^31^ demonstrated that overestimated values of hmF2 would shift the profiles upward, which could also create the vertical residual shape seen in the current study in Fig. 4. We have also investigated the performance of the IRI-2001corr topside parametrization. It was found that the vertical residuals also exhibited overestimation around 100-200 km above the F2-peak, which was larger than for the NeQuick topside ($\sim$60% compared to $\sim$40%). It is evident that the topside specification of the IRI has greatly improved over the years. At altitudes $\sim 150$ km above the peak, the mistakes decreased from $\sim$60% to $\sim$40%, while the main improvement by using the NeQuick option is achieved in the upper topside, where the errors reduced from $>20$% to a few percent.

The NET model was found to give unbiased predictions of the topside ionosphere based on the COSMIC data. In particular, at altitudes around 150 km above the F2-peak, which correspond to the largest bias of the IRI-2016, the NET model only exhibits errors in the order of a few percent. Furthermore, we analyzed the seasonal behavior of the models for different local times and magnetic latitudes (Supplementary Figure S4). We calculated the skill score values, which highlighted the regions where the NET model outperformed the IRI. The strongest improvement over the IRI (up to 80$\%$) was achieved in the local-winter hemispheres during solstices, and in both EIA crests during equinoxes. Furthermore, we performed model testing on 3 independent data sources, namely the GRACE-KBR, C/NOFS- Coupled Ion Neutral Dynamics Investigation (CINDI) and CHAMP- Planar Langmuir Probes (PLP) observations. The model was found to give highly accurate predictions on all of these independent data sources, reproducing even the fine structures of the ionosphere including the midlatitudinal ionospheric trough. In fact, over 90% of the NET predictions lie within a factor of 2 from the observations, both in comparisons with radio occultation and in situ measurements. It should be noted that several existing ML-based models had previously been tested on similar data sets. For instance, Gowtam et al.^16^ tested the ANNIM-3D model on the CHAMP-PLP data and observed correlation ranging from 60% to 79%, based on different locations, while for the NET model the correlations evaluated on the CHAMP data exceeded 90% (Table 1).

The NET model is based on observations from the COSMIC mission, which span at altitudes from hmF2 up to around 800-850 km altitude. The linear alpha-Chapman approximation can resolve electron density profiles up to around 1500 km in altitude^32^, and therefore our results can be used to reconstruct electron densities up to that altitude. Furthermore, it is possible to connect the model presented here to the plasmaspheric altitudes. Recently, Prol et al.^33^ demonstrated that the scale height in the plasmasphere exhibits a quadratic dependence on altitude, and found a functional dependence which allowed to connect the radio occultation profiles with in situ observations of electron density in the plasmasphere made by the Van Allen Probes mission. Using the methodology of Prol et al.^33^, it is possible to connect the model presented in this study to the plasmasphere, making it a full topside option valid up to the GNSS altitudes and beyond. Therefore, the model developed in this study combined with its plasmaspheric extension can be incorporated into the IRI as a novel option for specifying the topside ionosphere and plasmasphere.

## Conclusions

In this study, we developed a new empirical model of electron density in the topside ionosphere (NET) based on neural networks. The model uses the geographic and geomagnetic coordinates, local time, day of year, and solar and geomagnetic indices to predict the 4 parameters of the linear alpha-Chapman equation, namely the F2-peak density and height, and 2 parameters of the linear decay of scale height with altitude. The model has been trained and tested on $\sim 19$ years of radio occultation data and undergone additional validation on in situ observations by 3 independent missions. The NET model gives highly accurate and unbiased predictions for a variety of geophysical conditions, and outperforms the current topside options included into the International Reference Ionosphere model by up to 80%, especially at altitudes $\sim 100-200$ km above the F2-peak and in the local-winter hemispheres. In fact, the NET predictions are within a factor of 2 from the observations $\sim$90% of the time. The NET model can have wide applications in ionospheric research, for instance, in wave propagation studies, for calibrating the new electron density data sets with unknown baseline offsets, for tomographic reconstructions in the form of a background model, as well as to analyze specific space weather events and perform long-term ionospheric reconstructions. Furthermore, the developed model can be connected to plasmaspheric altitudes and thus can become a novel topside option for the IRI. The developed framework allows the seamless incorporation of new data and new data sources. The retraining of the model can be done on a standard PC and can be performed on a regular basis. Another contribution of this work can be incorporation of measurement uncertainty into the training which will allow inclusion of a variety of data sources with various observational errors.

## Methods

### Data set

Over the last two decades, the GNSS radio occultations proved to be an invaluable tool in the ionospheric research. The RO measurements are a remote sensing technique that allows retrieval of the high-resolution electron density profiles. Schreiner et al.^34^ estimated the precision of the RO observations to be $\sim$10$^3$el./cm$^{3}$. Currently, the electron density observations provided by the RO technique constitute a major three dimensional data source in the topside ionosphere. The EDPs are retrieved using an Abel inversion, which can lead to certain artifacts, for instance the underestimation of the EIA crests, arising from the underlying assumption of spherical symmetry^35^. However, the RO data have been extensively validated both in conjunctions with the ground-based ISRs and with satellite in situ observations. In particular, the high quality of the RO EDPs in the topside has recently been demonstrated by comparing the COSMIC measurements to electron density observations from the GRACE-KBR system^36^. The KBR data were calibrated by the ISRs^37^ and therefore were used as a reference data set for these comparisons. Smirnov et al.^36^ demonstrated that the radio occultation electron densities from the COSMIC mission were in very good agreement with GRACE data with bias of <2%. The study has shown that the RO observations can thus be an important data source for empirical modeling, especially in the topside ionosphere due to their 3D coverage and large volumes of provided data.

Several constellations have provided EDP data using the radio occultation technique, starting from the early days of the GPS/MET satellite. In this study, we use data from the COSMIC, CHAMP and GRACE missions. The details of spatial and temporal coverage of these missions can be found in Smirnov et al.^36^. In general, the COSMIC mission has provided an enormous data set of topside electron densities, exceeding 4.5 million profiles. However, the COSMIC mission operated during the declining phase of the solar cycle 23 and during the entire cycle 24 which corresponded to historically low levels of activity. The GRACE and CHAMP missions, on the other hand, also covered more active conditions during solar cycle 23. Therefore, in order to ensure a better solar cycle coverage and provide more data corresponding to active conditions, we include data from the GRACE and CHAMP missions. It should also be noted that these missions operated at much lower altitudes than COSMIC and do not provide enough coverage of high altitudes to fit the topside EDPs. Therefore, the additional GRACE and CHAMP data were used for retrieving NmF2 and hmF2, while the topside shape parameters were fitted on COSMIC EDP observations. In this study, we use level 2 electron density profiles, provided through the “IonPrf” product. The COSMIC EDP data were subject to quality control. We removed the topside profiles that extended over $>5^\circ$ GLat and $>10^\circ$ GLon, as well as EDPs that exhibited positive electron density gradients at higher altitudes. Furthermore, the profiles where the derivatives of electron density exceeded the magnitudes of electron density, and the profiles with deviations from the linear alpha-Chapman fit of more than 100% were removed, in order to exclude irregular EDPs.

The developed model needs to be validated not only on the data from the missions that were used for training, but also on independent data sources. Using independent missions for validation gives a good indication of the generalization ability of the model at different altitudes, locations and timescales. In this study, we employ the GRACE-KBR observations, which provide in situ electron density values with a spatial resolution around 200 km along the GRACE orbit. This dataset has been intercalibrated with incoherent scatter radars and can be considered as a “golden standard”^37^. Furthermore, we use in situ measurements of electron density by the CHAMP-PLP and the full ion densities measured by the C/NOFS-CINDI instrument, intercalibrated by Smirnov et al.^36^. The GRACE mission operated in 2002–2017 and covered altitudes from 400 to 500 km with global coverage. The CHAMP mission covered lower altitudes, from around 400 km at the beginning of the mission lifespan to 300 km at the end of the mission. The C/NOFS mission represents an important source of data for model validation, because it covered equatorial latitudes and also provided a vast altitude coverage from 300 km up to 800 km.

### Fitting the topside profiles with a linear alpha-Chapman function

The vertical structure of the ionosphere has been a topic of continuous interest since the early works of Appleton and Beynon^38^. The study by Bent et al.^25^ was one of the first papers that described the functional dependence of electron density in the topside as a combination of a parabolic and exponential terms. Since then, a variety of mathematical descriptions of the topside ionosphere has been developed. A systematic review of the possible functions to fit the topside profiles was presented by Fonda et al.^39^. In particular, they showed that the Chapman function gave the best agreement with observations based on the topside sounder data. The Chapman function is based on first principles and has been employed in numerous studies to approximate the topside ionosphere. One of the important parameters of the ionospheric plasma, included in the Chapman equation, is the effective scale height, which represents a vertical distance over which the electron density decreases by a factor of *e*^1^ and thus serves as a shape factor of the topside profiles.

The part of the topside ionosphere close to the F2 layer peak is dominated by atomic oxygen, while at higher altitudes the light ions become the dominant plasma constituents^22^. Different ion species have different scale heights, and this information needs to be incorporated into the empirical models. In the early works, these differences were neglected and the scale height was often assumed constant for simplicity^40^. This approximation works well in the lower topside (around the F2-peak) but faces obvious problems at higher altitudes. There can be several approaches to account for the varying scale height. One reasonable approach is to use a multi-layer model, for example assuming a Chapman function with a constant scale height near hmF2 but adding an exponential term for higher altitudes^41^. Another method, first proposed by Rishbeth and Garriott^1^, is to use a single layer formulation which includes an empirical relation of scale height to altitude. One of the approximations which gained a significant popularity assumes a linear decay of scale height with altitude^1,21,32,42–44^, and in this study is referred to as the linear alpha-Chapman function. It has been demonstrated that this method produced results largely identical to the multi-layer model and could well approximate both the radio occultation data^43^ and the topside sounder observations up to the altitude of around 1500 km^32^.

In this study, we approximate the COSMIC radio occultation profiles using the linear alpha-Chapman function of the form:

1 $$\left\{ {\begin{array}{*{20}l} {N_{e} (h) = {\text{NmF2}} \cdot \exp \left( {0.5(1 - z - \exp ( - z))} \right),} \hfill \\ {\;\;\;\;\;\;\;z = \frac{{h - {\text{hmF2}}}}{{H_{s} (h)}},} \hfill \\ {H_{s} (h) = \frac{{{\text{dHs}}}}{{{\text{dh}}}}(h - {\text{hmF2}}) + {\text{H}}_{{\text{0}}} ,} \hfill \\ \end{array} } \right.$$

where $N_e$ is electron density as a function of altitude *h*; NmF2 and hmF2 represent the peak electron density of F2-layer and the altitude of the peak, respectively; $H_s$ is an effective scale height, in our case depending linearly on altitude, $\mathrm {dH_s/dh}$ and $\mathrm {H_0}$ show the slope and intercept of this linear trend, respectively. The F2-peak density and height were obtained from the data, while the $\mathrm {dH_s/dh}$ and $\mathrm {H_0}$ values were retrieved by fitting the Eq. (1) to COSMIC EDPs using curve-fitting routines implemented in the Python scipy library.

Figure 1(b,c) shows an example of the COSMIC profile, fitted using the linear alpha-Chapman function. In panel (b), the COSMIC data are shown in orange, a large black dot denotes the peak of the F2-layer and is determined from the data, and the solid black line gives the topside profile fitted to Eq. (1). In panel (c), we demonstrate the scale height retrieved from the data using the method of Olivares-Pulido et al.^43^, and the corresponding linear fit from Eq. (1). In Fig. 1(b) it can be seen that the linear approximation reproduces the COSMIC profile well. In fact, Olivares-Pulido et al.^43^ showed that this functional dependence can approximate the vast majority of RO profiles with a correlation of over 98%. We note that the topside profiles are usually fitted to the linear alpha-Chapman method at altitudes higher than 100 km above hmF2, since the scale height may exhibit a stronger nonlinear decrease when approaching the F2-peak^21,43^.

The linear alpha-Chapman approximation yields 4 parameters, namely the F2-peak density and height, and 2 parameters of the linear scale height decay. In Fig. 1(d), we show the schematics of the model workflow. Each of the four parameters is modeled separately with a feedforward neural network, and the final values of electron density can be retrieved by substituting outputs of the 4 sub-models into the Eq. (1). It should be noted that NmF2 is predicted in logarithmic scale, due to the fact that the peak electron densities can span over several orders of magnitude, while the other parameters are retrieved in linear scale.

### Neural networks

One of the most efficient ways to utilize large volumes of data for empirical modeling is by using machine learning. In particular, artificial neural networks (NNs) are one of the most popular techniques to find complex non-linear relationships between the input and output variables, and have been used in many applications such as classification, regression, and image recognition. In this study, we employ multi-layer perceptrons (MLPs), which are a type of the fully connected feedforward neural networks. MLPs try to find nonlinear mappings between the input and output parameters by optimizing weights and biases of the neurons contained in the hidden layers. The neural network typically comprises an input layer, one or several hidden layers, and an output layer. Every link between a neuron in one layer to each of the neurons in the next layer has a corresponding trainable weight. It shows how strongly that particular neuron influences nodes it is connected with. When the network is initialized, the weights are selected randomly. Each connection also has an associated bias which allows to better adjust the model. At first, the values contained in the input layer nodes are summarized, passed through an activation function which adds a non-linearity into the model, and used as inputs to the neurons of the first hidden layer. Activation functions are an essential part of MLPs, as only with them one can model non-linear functions. This procedure is the same for all hidden layers. Finally, the outputs of the last hidden layer are summed up and given to the output node, usually without a final activation function. The weights and biases of each layer and neuron are typically optimized by gradient-based methods, where stochastic gradient estimates can be efficiently computed using back-propagation schemes.

Due to the expressiveness introduced by the large number of trainable parameters, MLPs can easily overfit to training data without capturing the underlying phenomena. One way to mitigate this is via regularization techniques, for instance the Dropout method^45^, which randomly removes a subset of neurons in training of each minibatch and adds regularizing effect by increasing the stochasticity. Another frequently employed technique is an addition of gaussian noise between the layers during the network training. It has been shown that adding small amounts of noise during the training process helps a NN converge to a smooth function of the inputs and also imposes a regularization effect. Specifically, the networks trained with additional noise are less able to memorize the training set, as the model perceives the training samples infused with noise as constantly changing. This results in more robust networks with lower generalization errors. In this study, we employ both the gaussian noise and dropout techniques and treat the dropout rate and noise magnitude as hyperparameters.

In the present study, we develop a set of models to predict 4 parameters that can be represented as numerical variables and therefore address a regression problem. As inputs, we use the solar flux index P10.7, geomagnetic indices Kp and SYM-H, as well as satellite position in geographic and geomagnetic coordinates, MLT and day of year (DOY). It is of note that the COSMIC EDPs can span over several degrees in latitude and longitude, and therefore the geographic positions of the top points of the profiles were used to train the $\mathrm {dH_s/dh}$ and $\hbox {H}_0$ models, while during the model testing the profiles were assumed vertical. The P10.7 represents a smoother version of the 10.7 cm radio flux index (F10.7), and is derived as an average of the current F10.7 value and that over the previous 81 days^46,47^. This index has been used in a variety of ionospheric models, including the IRI model, and was found to give better performance than the raw F10.7 values^48^, which can exhibit spikes, especially during the strong solar storms. We also use the planetary Kp index that shows the averaged state of the geomagnetic field disturbances and serves as a good proxy of convection which is an important mechanism in producing several ionospheric phenomena, for instance, the polar patches. Furthermore, we use the SYM-H index, which shows the strength of the geomagnetic storms, to account for the storm-time events.

Several of the cyclic input features have artificial boundaries between the highest and lowest values. For instance, the local time has a discontinuity at 24-00 hours, which can create artifacts in the model output. In order to avoid this discontinuity, it is common practice in empirical modeling to replace values of these input features with their sine and cosine values^49^. Furthermore, it has been shown that using higher orders of the sine and cosine functions for the positional inputs can significantly increase the model accuracy. Tancik et al.^50^ demonstrated that these simple feature transformations allowed the MLP models to learn the high-frequency dependencies in low-dimensional regression tasks and greatly enhanced the model performance for image regression problems. This technique became known as the Fourier features method^50^. In this study, we also apply this method to several features, namely the LT, DOY, geomagnetic and geographic latitude and longitude (see Fig. 1d). Selecting the correct FFT order can have a non-negligible effect on model training. For each of the features, we select the FFT order that results in the best model performance (the corresponding selection of the FFT orders is described in the Supplementary information).

### Data splitting

This study uses a supervised learning algorithm, namely a multilayer perceptron, to model electron density in the topside ionosphere. In order to train the supervised model, it is necessary to split the data into the training, validation and test subsets. The training set is used to fit the model, the validation set helps select the neural network hyperparameters and gives a more unbiased estimate of the network performance during each training iteration. In particular, the error on the validation set can be used for early stopping regularization, where the model training terminates as soon as the validation error does not decrease anymore. The test set is withheld during the model training and validation, and is only used once the models have been created to evaluate their generalization ability on the unseen data.

There are several ways to split the data into the training, validation and test data sets. If no time-dependence is assumed in the data, it is possible to split data entries between the three sets randomly, e.g., by using 70-80% of the data for training, and 10% for both validation and testing. In case of time-series and physics problems, this splitting technique should be avoided as it can lead to what is referred to as the data leakage. For instance, when most of the points constitute the training set, one can linearly interpolate between those points to derive values that belong to the typically smaller validation and test sets. This would result in a biased estimate of the model performance^51,52^. Another potential technique is to use the K-fold cross validation (CV), where the data are split in the time-domain into K continuous parts, and the model is re-fitted K times each time withholding one of the parts and trained on K–1 intervals (for details, see e.g., Smirnov et al.^52^). At the same time, using the K-fold CV for hyperparameter tuning leads to an exponential increase in training time, as the models need to be re-trained numerous times for every hyperparameter trial. Therefore, another option is to split the data in the time domain into a number of intervals that are long enough to contain independent events of shorter timescales, therefore preventing the data leakage. This is described, for instance, in Chu et al.^53^. In this study, we split the data into continuous 27-day segments, and randomly choose 70% of those intervals for training, 15% for validation and 15% for testing. Such a splitting allows us to validate the model on a variety of solar activity and geomagnetic conditions, while at the same time avoiding the data leakage. The data splitting is illustrated in Fig. 1(a), where one colored stripe corresponds to a single 27-days segment.

### Hyperparameter tuning/ model selection

In this study, we use feedforward neural networks implemented in the Keras Python library^54^. The parameters which define the network structure and training procedures are typically referred to as the hyperparameters and include the number of the hidden layers, number of neurons in each of the layers, the activation function, optimizer, dropout rate and so on. They are not directly optimized by gradient descent. Instead, we use the tree-structured Parzen estimator algorithm^55^ implemented in the Optuna Python library to tune the number of hidden layers and neurons, dropout rate and the magnitude of gaussian noise. The preliminary hyperparameter trials indicated that a 3 layer neural network achieved a very good performance and the reduction of the MSE by adding the fourth and fifth layers was <1 %. Therefore, we later fixed a number of layers to 3 and optimized the other parameters. The summary of the parameters, their search domains and the optimized values based on the Optuna trials are given in the Supplementary Table S1.

### Comparison with the International Reference Ionosphere (IRI) model

First established as a joint project of the Committee on Space Research (COSPAR) and International Union of Radio Science (URSI) in 1968^56^, the International Reference Ionosphere is, perhaps, the most famous model of the ionosphere. The IRI has been continuously improved^10^ and in 2014 was accepted by the International Standards Organisation (ISO) as the international standard of ionospheric specification^6^. The IRI describes electron density and temperature, ion composition, ion temperature and drifts at altitudes from $\sim$50 up to around 2000 km. For electron density modeling, NmF2 and hmF2 can be considered the most important parameters. There are several options for the topside shape functions, including the IRI-2001 version and its correction^57^, as well as the topside parametrization based on the NeQuick model^7,8^. Since the 2007 version of the IRI, the NeQuick topside has been adopted as the default option for the topside ionosphere^9^. In this study, we use the IRI-2016 model with the foF2 specified by the URSI model, hmF2 given by AMTB-2013 model^58^, and the default NeQuick topside, to compare to the predictions by the developed NET model based on several in situ and radio occultation data sets. In particular, it is crucial to quantify the degree of improvement achieved by the NET model compared to the IRI, which can be done using the skill score (SS) metric. This metric can be written as follows:

2 $$\begin{aligned} SS = 1 - \dfrac{\sum _{i=1}^N (m_i - o_i)^2}{\sum _{i=1}^N (b_i - o_i)^2}, \end{aligned}$$

where *m* denotes the NET model values, *o* stands for observations, and *b* represents the baseline (IRI) model output. This metric quantifies the improvement over a baseline model, and is also sometimes referred to as the prediction efficiency (PE)^59,60^. The skill score values are analyzed in detail in the Supplementary information for different seasons, magnetic latitudes and local times, and it is demonstrated that the NET model outperforms the IRI-2016 by up to 70–80%, with the most significant improvement achieved in the local-winter hemispheres (Supplementary Figure S4).

## Supplementary Information

Supplementary Information.

## Data Availability

The data and model files and example codes are available at https://doi.org/10.5880/GFZ.2.7.2023.001, and the latest model updates are provided on https://github.com/arsmirnov95/NET_topside_model. The CHAMP, GRACE and COSMIC radio occultation data were obtained from University Corporation for Atmospheric Research (UCAR) through the COSMIC Data Analysis and Archival Center (CDAAC) via the portal https://cdaac-www.cosmic.ucar.edu/.
