# Supplementary material for: A novel neural network model of Earth’s topside ionosphere
Source: Sci Rep. 2023 Jan 24;13:1303. doi: 10.1038/s41598-023-28034-z (PMC9873638; doi:10.1038/s41598-023-28034-z)
Supplement: Supplementary file 1 — Supplementary Information. [file 41598_2023_28034_MOESM1_ESM.pdf]

Supplementary Information for "A novel neural network model of Earth's topside ionosphere"

Artem Smirnov[1,2,*], Yuri Shprits[1,2,3], Fabricio Prol[4,5], Hermann Lühr[1], Max Berrendorf[6], Irina Zhelavskaya[1], and Chao Xiong[7]

[1]Helmholtz Centre Potsdam - GFZ German Research Centre for Geosciences, Potsdam, Germany
[2]Institute of Physics and Astronomy, Potsdam, Germany
[3]Department of Earth, Planetary and Space Sciences, University of California Los Angeles, CA, USA
[4]Finnish Geospatial Research Institute (FGI), National Land Survey of Finland (NLS), Department of Navigation and Positioning, Kirkkonummi, Finland
[5]Institute for Solar-Terrestrial Physics, German Aerospace Center, Neustrelitz, Germany
[6]Institute of Informatics, Ludwig-Maximilians-University of Munich, Munich, Germany
[7]Department of Space Physics, College of Electronic Information, Wuhan University, Wuhan, China
[*]artem.smirnov@gfz-potsdam.de

Table S1. Neural network (NN) hyperparameters, their search domains and the optimized values for the four models.

Hyperparameter	Search range	NmF2	hmF2	H_0	dH_s/dh
# neurons in the 1st layer	4–1024	256	256	256	256
# neurons in the 2nd layer	4–1024	512	64	64	64
# neurons in the 3rd layer	4–1024	64	256	256	256
dropout rate	0–0.3	0.05	0.139	0.008	0.1
gaussian noise σ	0–0.3	0.17	0.0713	0.0004	0.19
learning rate	10^{-6}–10^{-2}	0.0004	0.0011	0.000443	0.00037
batch size	16–128	32	64	128	32

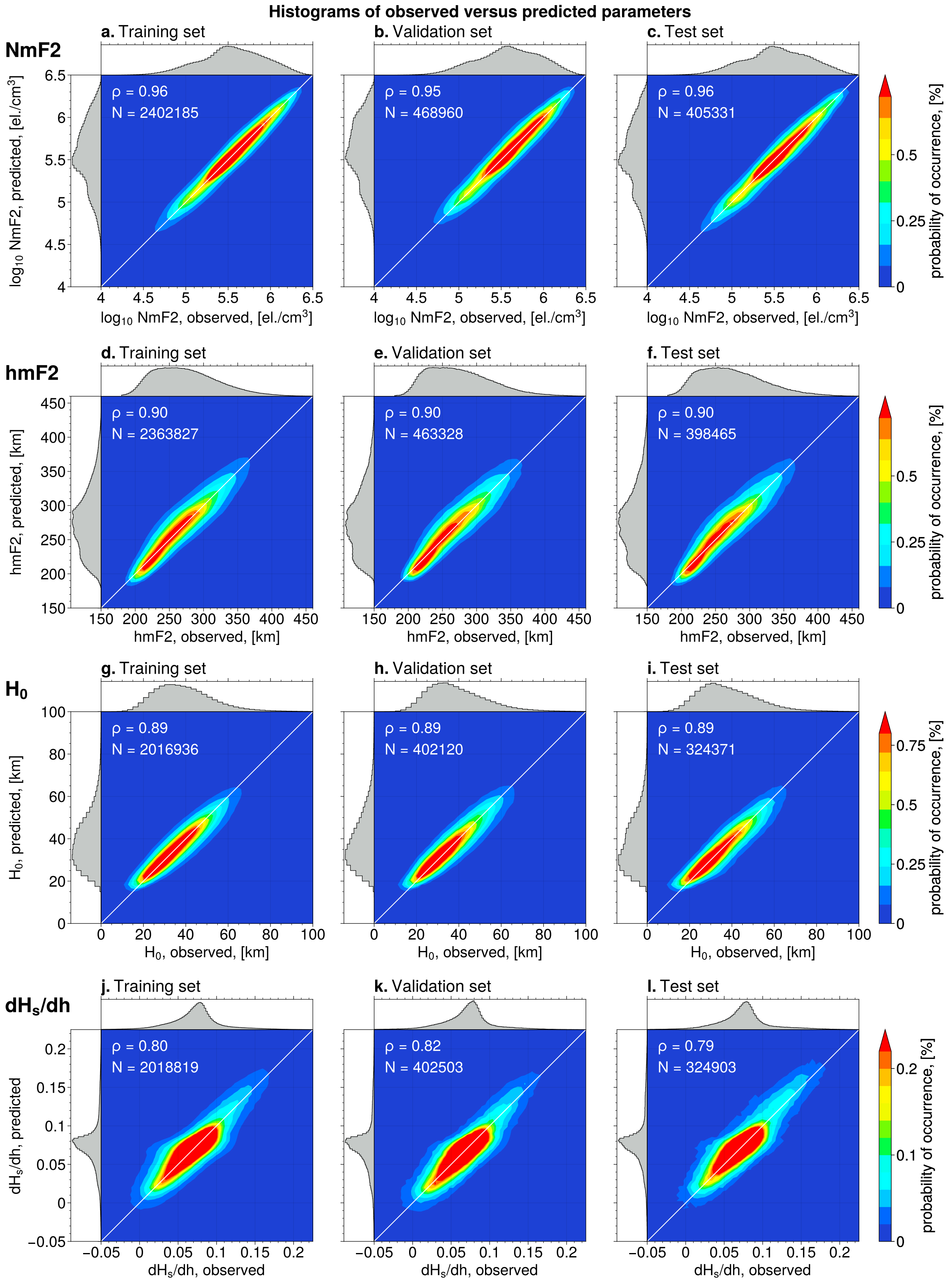

Figure S1. 2D histograms of observed versus predicted parameters (NmF2, hmF2, H_0 and dHs/dh) on the training, validation and test sets. Each subplot gives values of the Spearman rank correlation (ρ) and the number of points (N). The one-to-one correspondence lines are shown in white. One can see that the NET model reproduces all of the parameters well, with correlations ranging from 0.8 up to 0.96.

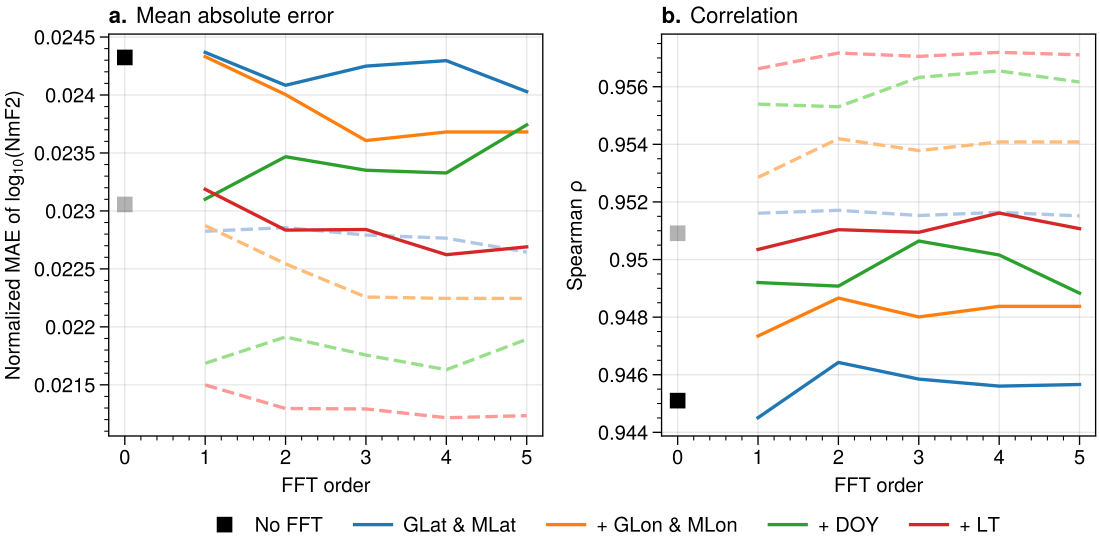

Figure S2. Selection of FFT order for different input features on the example of NmF2 sub-model. The solid lines in the Figure show MAE and correlation values on the validation set, while the shaded dashed lines give values on the training set. We start from the "base" model that does not contain any FFT features but only the untransformed values of the inputs. We then replace the geographic and magnetic latitudes with sine and cosine values of different orders, up to order 5. It is of note that the MAE values were almost identical for orders 2 and 5, but the correlation was slightly higher for the second order, and therefore it was selected. To this model, we then add FFT features of geographic and magnetic longitude, and select order 3 which results in the smallest MAE. The same procedure is repeated for the day of year and local time, and select orders 3 and 4, respectively. The overall MAE of the final model is reduced by 7% compared to the initial base model that does not use FFT features.

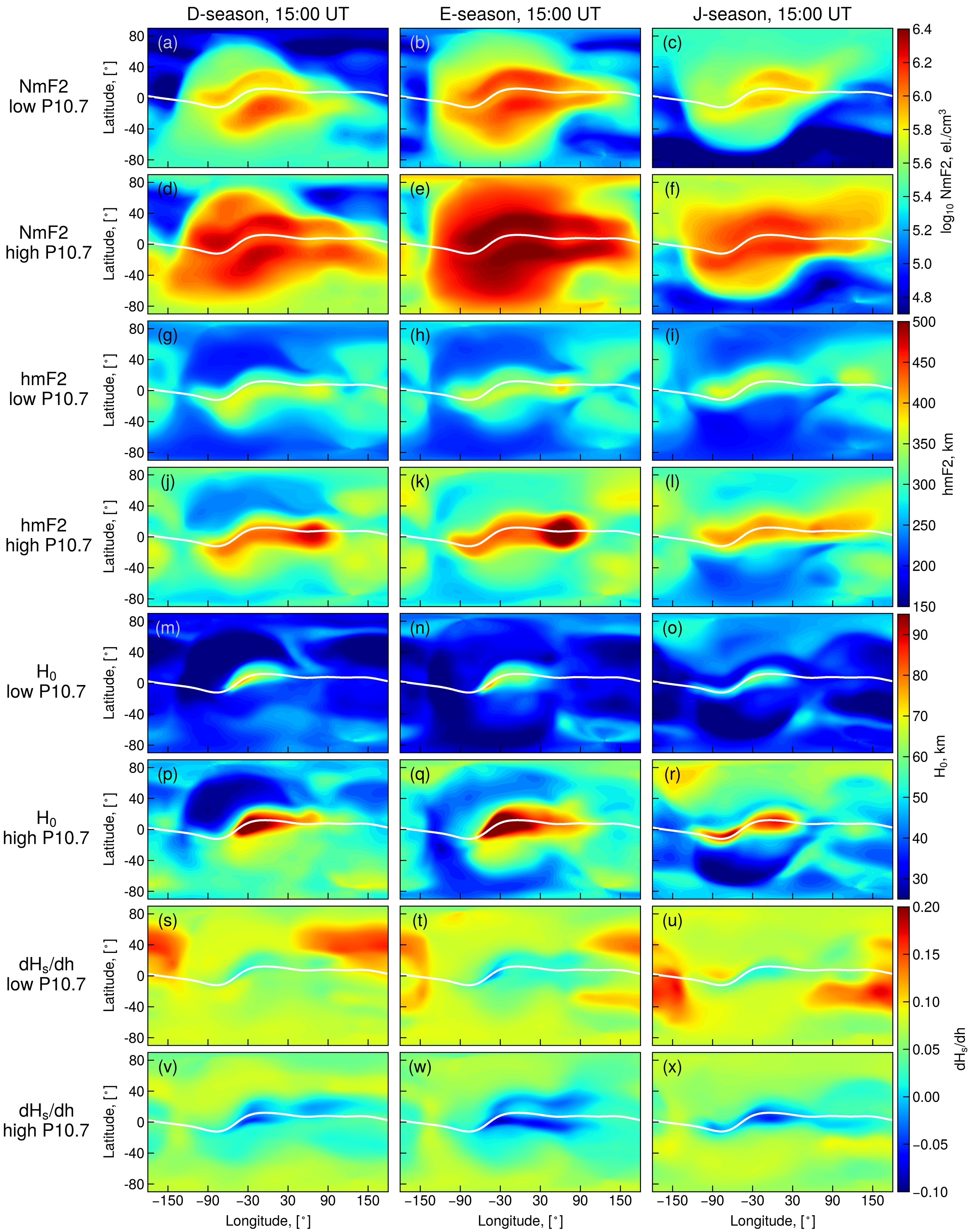

Figure S3. Synthetic runs of the NET model for different seasons under high (P10.7=180 sfu) and low (P10.7=80 sfu) solar activity. The solid white line shows the magnetic equator.

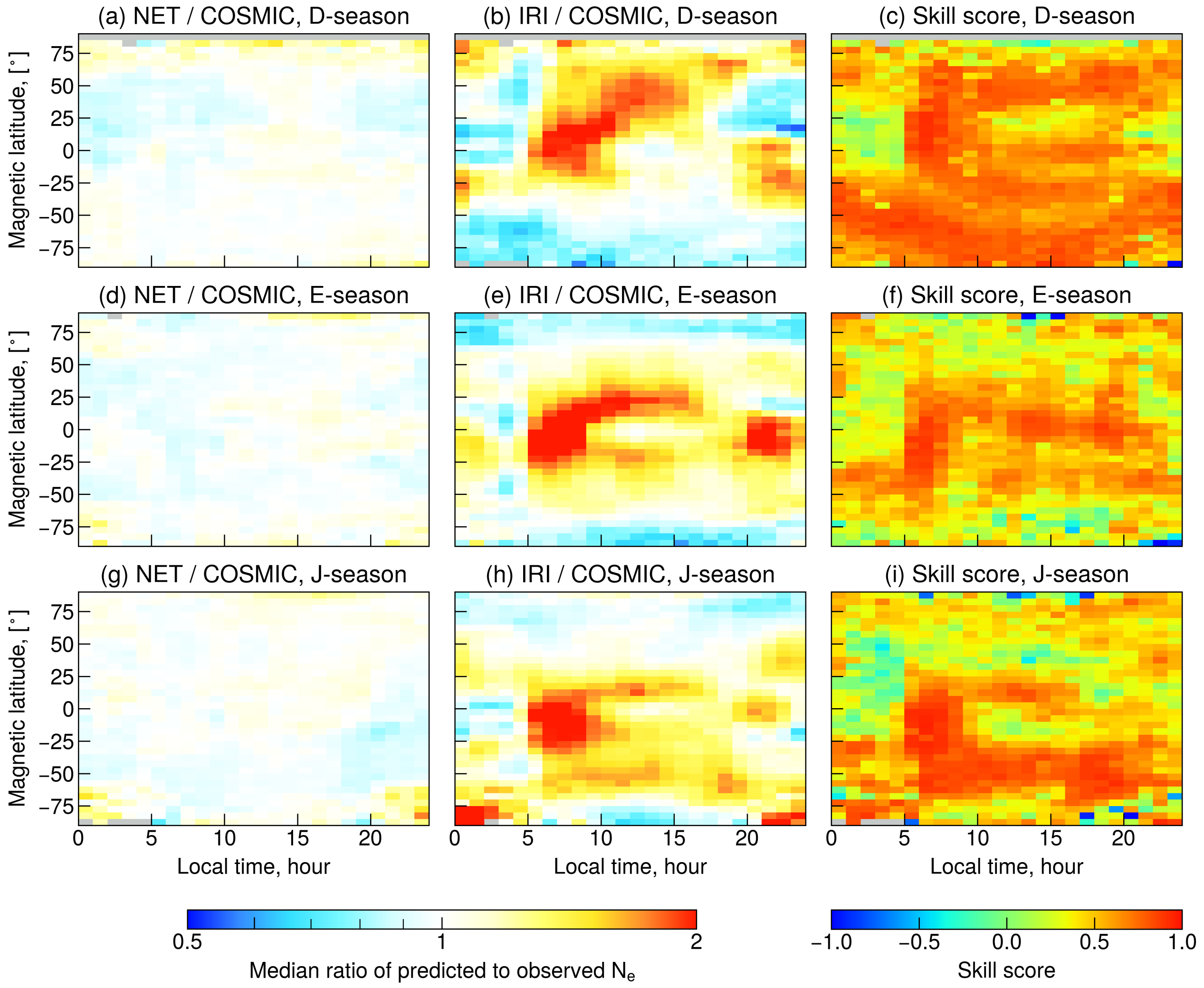

Figure S4. A comparison between the model predictions to COSMIC data on the test set for different seasons, binned by magnetic latitude (MLat) and local time (LT). Median ratios of the NET predictions to COSMIC electron densities are shown in the left column. The ratios are generally close to 1 at all MLats and LTs both for the equinoctial and solstice conditions. The middle column shows the median ratios between the IRI and COSMIC densities. The IRI exhibits overestimation of the local-winter hemispheres at mid-latitudes during solstices and overestimates the EIA crests during equinoxes. The column on the right shows the associated skill score values, with higher values correspond to regions where the NET model outperforms the IRI. One can see that the improvement of the NET model over the IRI is up to ~80%.

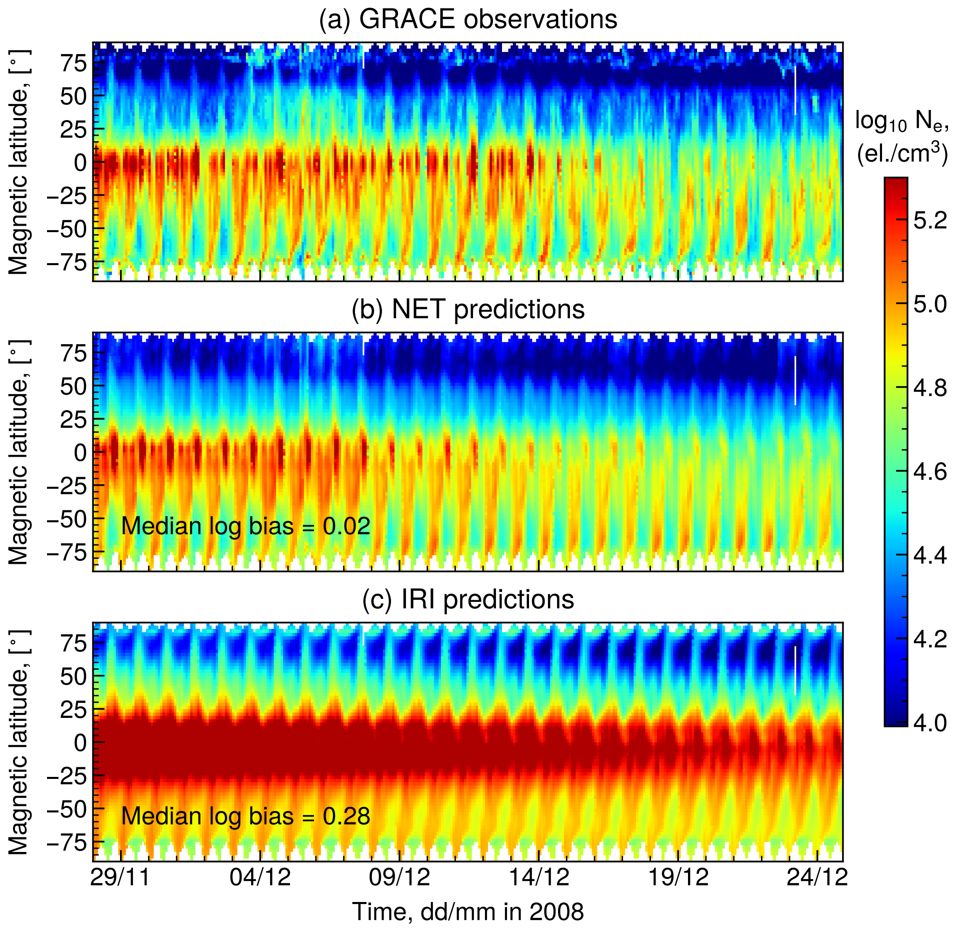

Figure S5. A comparison between the NET and IRI predictions to independent data from GRACE-KBR based on a 27-day period from the test set. (a) GRACE-KBR electron densities, (b) NET predictions, (c) IRI predictions. The NET predictions agree with GRACE data very well and reproduce even the fine structures of the ionosphere such as the midlatitude trough (MLat$\sim$60°), which is not captured by the IRI.

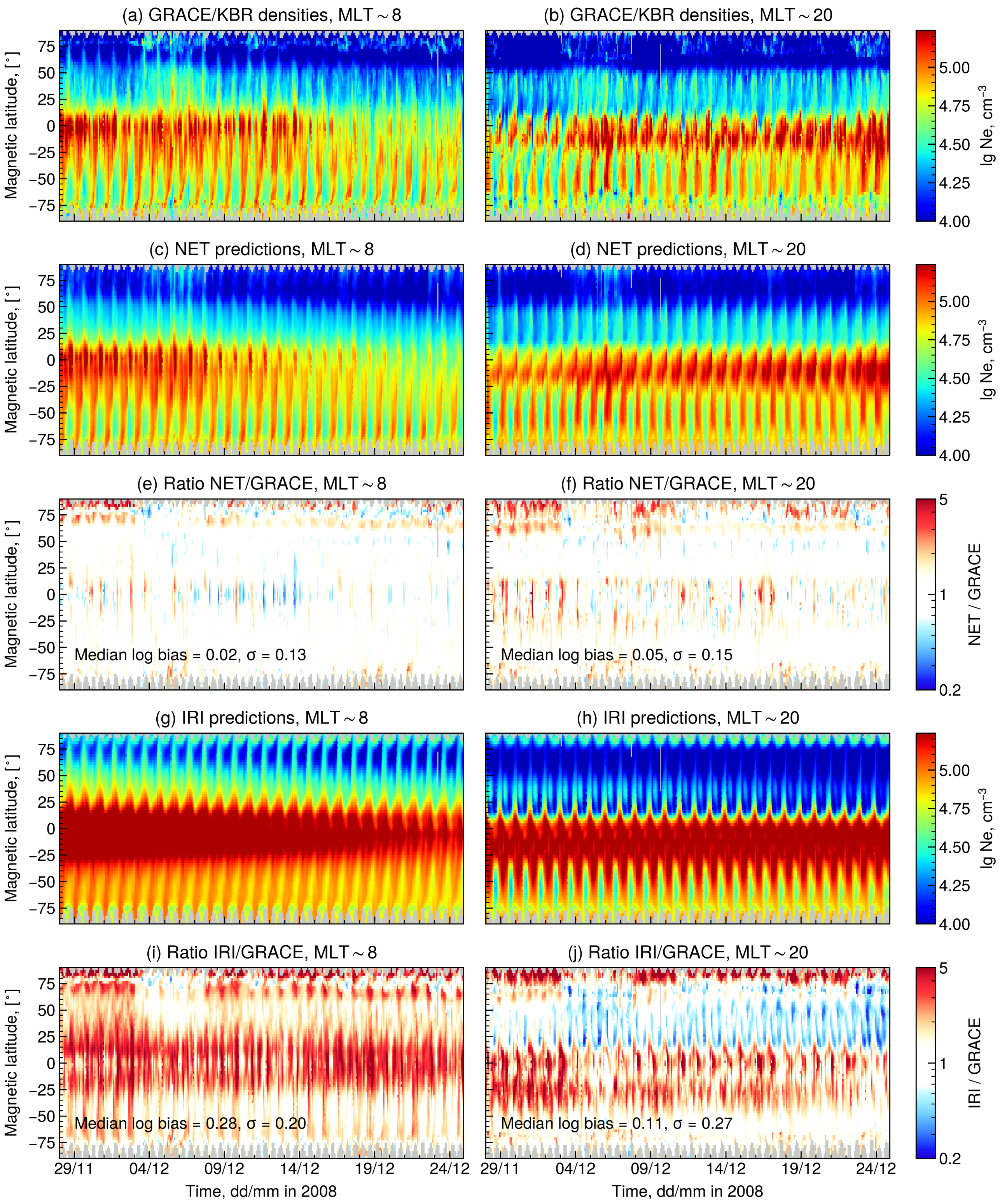

Figure S6. A comparison between the NET and IRI models to independent data from GRACE-KBR based on a 27-day period from the test set. (a,b) GRACE-KBR electron densities for rising and setting satellite passes, (c,d) NET predictions, (e,f) ratios between the NET and GRACE electron densities. Panels (g,h) show the IRI predictions and panels (i,j) give the ratios between IRI and GRACE. One can see that The NET predictions agree with GRACE data very well, with ratios close to 1.

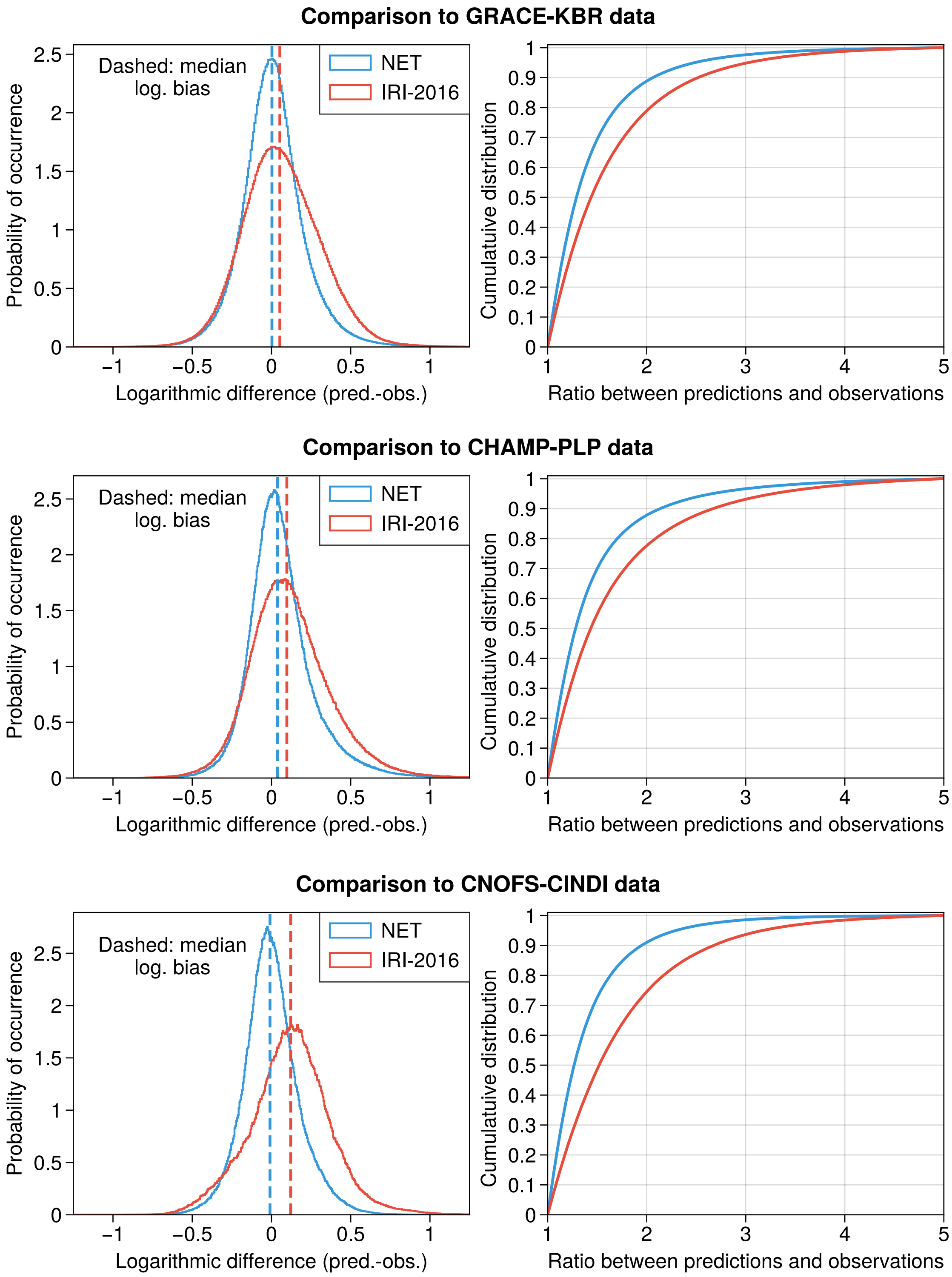

Figure S7. Statistical comparisons of the predictions by the NET and IRI models to independent observations from CHAMP, GRACE and C/NOFS missions. The left column demonstrates histograms of the logarithmic residuals for both models. The right column gives the cumulative distributions of the ratios between models and observations (similar to Figure 3 in the main text). The NET model gives unbiased predictions on all 3 independent data sets, as the bias is very close to 0. Furthermore, the NET predictions are within a factor of 2 from the observations $\sim$90% of the time. This is highly consistent for all missions, and agrees well with comparisons to RO data shown in Figure 3 in the main text. Therefore, the developed NET model not only reproduces the radio occultation profiles which it was trained on, but also shows a remarkable agreement with fully independent data sources during time periods that were not used for model training.
